# Efficient White Electrochemiluminescent Emission From Carbon Quantum Dot Films

**DOI:** 10.3389/fchem.2020.580022

**Published:** 2020-09-29

**Authors:** Jonathan Ralph Adsetts, Ruizhong Zhang, Liuqing Yang, Kenneth Chu, Jonathan Michael Wong, David A. Love, Zhifeng Ding

**Affiliations:** ^1^Department of Chemistry, The University of Western Ontario, London, ON, Canada; ^2^Tianjin Key Laboratory of Molecular Photoelectronic Sciences, Department of Chemistry, Tianjin University, Tianjin, China; ^3^Rosstech Signal Inc., Orillia, ON, Canada

**Keywords:** carbon quantum dots, electrochemiluminescence (ECL), light emitting electrochemical cell (LEC or LEEC), lighting, half cells

## Abstract

Carbon quantum dots (CQDs) were manufactured from citric acid and urea in a gram-scale synthesis with a controlled size range between 1. 5 and 23.8 nm. The size control was realized by varying volume of the precursor solution in a hydrothermal synthesis method. The prepared CQDs were investigated using electrochemiluminescence (ECL) spectroscopy at interfaces of their electrode films and electrolyte solution containing coreactants rather than conventional optoelectronic tests, providing an in-depth analysis of light-emission mechanisms of the so-called half-cells. ECL from the CQD films with TPrA and K_2_S_2_O_8_ as coreactants provided information on the stability of the CQD radicals in the films. It was discovered that CQD^•−^ has a powerful electron donating nature to sulfate radical to generate ECL at a relative efficiency of 96% to the Ru(bpy)_3_Cl_2_/K_2_S_2_O_8_ coreactant system, indicating a strong performance in light emitting applications. The smaller the CQD particle sizes, the higher the ECL efficiency of the film interface, most likely due to the increased presence of surface states per mass of CQDs. Spooling ECL spectroscopy of the system revealed a potential-dependent light emission starting from a deep red color to blue-shifted intensity maximum, cool bright white emission with a correlated color temperature of 3,200 K. This color temperature is appropriate for most indoor lighting applications. The above ECL results provide information on the performance of CQD light emitters in films, permitting preliminary screening for light emitting candidates in optoelectronic applications. This screening has revealed CQD films as a powerful and cost-effective light emitting layer toward lighting devices for indoor applications.

## Introduction

Light emitting diodes (LEDs) have been at the forefront of lighting technology recently due to their decreased energy consumption over compact fluorescent and incandescent lighting technologies. LEDs are conventionally created by depositing many subsequent layers on a substrate in an inert atmosphere (Zhang et al., 2013; Gao, 2018; Kusamoto and Nishihara, 2018; Yang et al., 2019). Reducing the number of layers and removing the need for an inert atmosphere can drastically reduce manufacturing costs and operating voltages. To this end, light emitting electrochemical cells (LECs or LEECs) have gained interest. LECs conventionally comprise of two electrodes sandwiching a light-emitting layer that is responsible for both charge transport and light emission (Fresta and Costa, 2017). The light-emitting layer typically consists of a polymer and a salt with an incorporated light emitter which organizes itself into a p-i-n junction when an external bias is applied (Gao, 2018). The polymer electrolyte reduces bulk and contact resistance over LEDs, allowing for air-stable and thicker electrodes. The reduced potentials and simpler device structure allow for cost-effective manufacturing of energy efficient lighting sources.

Developing LEC technology as a consumer product requires research efforts into analyzing the light emitting layer for its polymer consistency, ion rearrangement efficiency, and electron and hole transportation for radiative recombination. Preeminently, a light emitter with poor electron and hole accepting properties will not efficiently radiatively recombine in an LEC, thus not producing substantial light. To understand the solid-state electron and hole transport mechanisms in light emitters, we propose using electrochemiluminescence (ECL) spectroscopy. ECL spectroscopy effectively allows electron and hole transport mechanisms to be studied independently in a light emitting material, as well as the efficiency and characteristics of the resulting radiative recombination for both electron and hole mechanisms. Studying these processes separately allows for a more fundamental understanding of the overall process of light emission from LECs, which allows the light emission to be improved upon.

An attractive light emitting material for use in LECs is carbon quantum dots (CQDs) due to efficient visible light emissions with tunable band gaps (Yuan et al., 2018; Qin et al., 2019; Chen et al., 2020a). First discovered in 2004 (Xu et al., 2004), CQDs are small sp^2^ and sp^3^ containing carbon particles that have been defined as having sizes below 20 nm, low toxicity, strong chemical stability and a resistance to photobleaching (Gan et al., 2016; Hu et al., 2019; Chen et al., 2020b). This study will use ECL spectroscopy to evaluate CQD's electron and hole transport mechanisms and the efficiency of radiative recombination for applications in optoelectronic. Further, light emission characteristics are reported and the suitability for indoor lighting applications will be assessed.

## Materials and Methods

### Chemicals and Materials

Citric acid (C_6_H_8_O_7_, 99%), urea (OCN_2_H_4_, 99%), potassium persulfate (K_2_S_2_O_8_, 99.99%), sodium phosphate monobasic dehydrate (NaH_2_PO_4_·2 H_2_O, ≥99%), and tris(2,2'-bipyridyl)-dichlororuthenium(II) hexahydrate [Ru(bpy)_3_Cl_2_·6 H_2_O, 97%] were purchased from Sigma-Aldrich (Mississauga, ON). Sodium phosphate (Na_2_HPO_4_, anhydrous, ≥99%) was obtained from Caledon Laboratory Chemicals (Georgetown, ON). Potassium chloride (KCl, 99%) was purchased from Alfa Aesar (Ward Hill, MA). Carboxymethylchitosan [(C_10_H_19_NO_6_)_n_, 99%] was obtained from Santa Cruz Biotechnology, Inc., (Dallas, TX). Ultrapurewater (18.2 MΩ cm, Milli-Q, Millipore) was used to prepare solutions. All chemical reagents were used as received and stored at room temperature with exception of carboxymethylchitosan stored at 4°C.

### CQD Synthesis Procedure

The following synthesis procedure was for CQD20, but all syntheses follow the same general format. 1.0 g citric acid and 2.0 g of urea were combined in 20 mL of Milli-Q water and sonicated for 10 min inside a 100 mL Teflon-lined autoclave were acquired from Shanghai Yuhua Instruments Equipment Co. Ltd, China. The steel autoclave used supports pressures up to 3 MPa. A VWR oven was set to warm up to and hold 160°C for 6 h, then cool down. After returning to room temperature, the autoclaves were removed from the oven and the solution was transferred directly into dialysis bags (Shanghai Yuanye Bio-Technology Co. Ltd, China) with a molecular weight cut-off (MWCO) of 1,000 Da. The solution was left to dialyze for at least 8 h, with the water being changed at least 6 times. The solutions were transferred to 50 mL Falcon tubes (VWR Canada), where a Kimwipe was attached to the top with an elastic band. This Falcon tube containing solution was frozen in liquid nitrogen and placed in a Labconco Lyophilizer at −84°C for at 48 h. The obtained product was light and fluffy and ranged from dark green to brown in color. These CQDs were stored in a refrigerator, sealed with Parafilm, and wrapped in tinfoil to prevent any degradation until use.

### CQD Characterization

High resolution transmission electron microscopy (HRTEM) images were obtained using a FEI Tecnai G2 F20 microscope. CQD powders were pressed in a sample holder of a FTIR spectrometer (VERTEX 70 FTIR) and measured. Background and blank measurements were taken before spectra acquisition to better identify peaks. UV-visible measurements were taken from 900 to 200 nm using a Varian Cary 50 Spectrophotometer (Varian Inc., North Carolina) where background and blank measurements were taken before for more accurate results. Photoluminescence (PL) measurements were taken with a Fluorolog spectrophotometer (QM-7/2005, Photon Technology International, London. ON) with excitation and emission slit widths of 0.25 and 0.1 mm respectively. All UV-Visible and PL measurements were done in a 10 mm quartz cuvette. The PL quantum yield (QY) was calculated using the following equation:

$$\begin{array}{l} \Phi_{\text{PL}}=\frac{I_{x}}{I_{st}}\;\frac{A_{st}}{A_{x}}{\left(\frac{\eta_{x}}{\eta_{st}}\right)}^{2}x\;100\;\% \end{array}$$

where *I* is the integrated PL emission intensity of an emission spectra excited at 350 nm, *A* is the absorbance value measured at 350 nm from the UV-Vis spectra, η is the refractive index of the solvent, *x* and *st* refer to the CQDs and the PL standard quinine sulfate in 0.1 M HCl (Eaton, 1988). 350 nm was used for quinine sulfate and the max excitation wavelength for each CQD was used for QY experiments, respectively.

### Electrochemical and ECL Experiments

A custom photoelectrochemical cell, with a flat Pyrex window at the bottom to allow the detection of light generated at the working electrode, was used for all electrochemical and ECL tests. A three-electrode electrochemical system, where a glassy carbon electrode (GCE, 3 mm diameter) was used as the working electrode, a platinum wire was used as the counter electrode and a custom Ag/AgCl electrode calibrated to an industrial standard Ag/AgCl electrode before operation. All solutions used were 0.1 M phosphate buffer solution (PBS) (pH = 7.5) with 0.1 M KCl as the supporting electrolyte. Dissolved oxygen in the system had a quenching effect for CQD ECL systems seen previously (Zhang et al., 2017), so all solutions were purged for 15 min with argon gas before use. For ECL film studies, 3 mg of GQDs were dispersed in 3 mL of Milli-Q water and were sonicated for 10 min before use. Ten Microliter of this solution was dropcasted onto the surface of the GCE and dried at room temperature. To prevent the GQDs dispersing in solution, a thin layer of Chitosan (0.2 mg mL^−1^ in Milli-Q water, 5 μL) was dropcasted on top of the GQD layer.

The voltammetric ECL curves were obtained using an electrochemical workstation (CHI 610A, CH Instruments, Austin TX) coupled with a photomultiplier tube (PMT, R928, Hamamatsu, Japan) held at −750 V with a high-voltage power supply. The ECL generated at the working electrode was collected by the PMT under the Pyrex window at the bottom of the electrochemical cell. The photocurrent from the PMT was transferred into a voltage signal by a picoammeter (Keithley 6487, Cleveland, OH). This signal, along with the potential and current signals from the electrochemical workstation were simultaneously sent through a data acquisition board (DAQ 6036E, National Instruments, Austin TX) to the computer where the entire data was recorded by a homemade LabVIEW (National Instruments) program. Spooling ECL spectra were acquired by placing the electrochemical cell into a holder on a spectrograph (Cornerstone 260 M, Newport, Irvine, CA) with a CCD camera (Andor 420BV, Andor Technology, UK) cooled to −55°C. The exposure time and the number of kinetic series were optimized to produce the clearest ECL spectra. A carefully measured lens system which collimated light produced from the entire electrode surface (~7 mm^2^ circle) onto the spectrometer/CCD camera set, permitting sensitive detection of light emitted from CQD films on the electrode surface while ignoring any other potential light sources. During all experiments, lights in the experimentation room were turned off to reduce the background interference from ambient light. Blackout curtains were also positioned at the entryways to the lab and surrounding the electrochemical cell setup to prevent possible interference. Wavelength calibration was conducted using a mercury-argon source (HG-1, Ocean Optics, Largo, FL). The relative efficiency of the ECL emission was calculated by finding the charge input and the photocurrent output for this specific experimental setup and comparing these values to the gold standard ECL emitter systems, Ru(bpy)${}_{3}^{2+}$ for annihilation systems and Ru(bpy)${}_{3}^{2+}$/S_2_O${}_{8}^{2-}$ for CQD/ S_2_O${}_{8}^{2-}$ systems by the following equation:

$$\begin{array}{l} \Phi_{\text{ECL}}=\frac{{\left(\frac{\int \text{ECL dt}}{\int \text{Current dt}}\right)}_{x}}{{\left(\frac{\int \text{ECL dt}}{\int \text{Current dt}}\right)}_{st}}\;x\;100\;\% \end{array}$$

where *st* and *x* refer to the standard Ru(bpy)${}_{3}^{2+}$/S_2_O${}_{8}^{2-}$ and the CQD/S_2_O${}_{8}^{2-}$ systems, respectively, for example.

## Results and Discussion

### Carbon Quantum Dot Synthesis

**Scheme 1:**
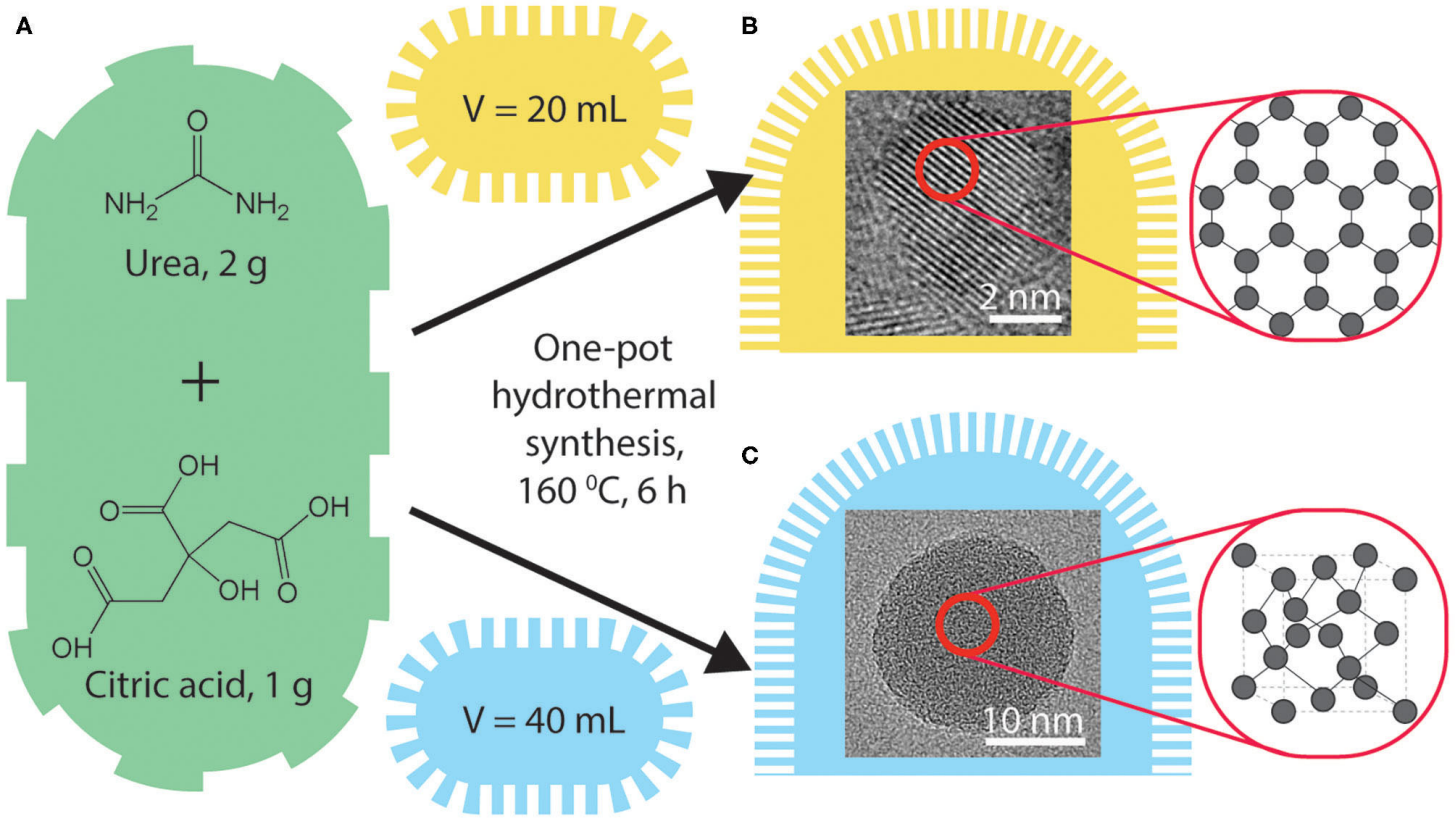
A schematic representation of CQD synthesis from precursors **(A)**. HRTEM images of the created CQDs with carbon arrangements identified, synthesized with autoclave volumes of 10, 20 mL **(B)** and 30, 40 mL **(C)**.

Citric acid was used as the carbon precursor for a hydrothermal synthesis owing to the efficient carbonization as reported by Dong et al. (2012). Urea was added as a nitrogen-dopant (N-doped) following our previous studies that report photoluminescence (PL) and ECL enhancements of nitrogen- and sulfur-doped graphene quantum dots (Zhang et al., 2017). Typical hydrothermal procedures were used as following: 50.0 g/L of citric acid and 100.0 g/L of urea in varying volumes of ultrapure water in a 100 mL Teflon-lined autoclave and heated at 160°C for 6 h. The volumes of the starting precursor solution were 10 mL (CQD10), 20 mL (CQD20), 30 mL (CQD30), and 40 mL (CQD40), respectively, as seen in Scheme 1. Yields of synthesis were measured by comparing the weight of starting precursors to the weight of the final CQD product and were found to be between 27 and 35%. An important factor for light emitting materials is the bulk low-cost synthesis of the product (Jing et al., 2019). This synthesis procedure created gram-scale CQDs with constant oven settings and constant starting solutions providing ideal conditions for scaling up synthesis of CQDs with tunable light emitting properties. A simple and gram-scaled synthesis is always required for optimization and testing of the CQDs' PL, ECL, and EL emission properties for future device testing. The cost and simplicity of CQDs described above make them an attractive light emitting material for future LEC devices over single molecules (such as highly luminescent carbozolyl derivatives Li et al., 2019), copolymers [e.g., commercially available PPV copolymer PDY-132 Gambino et al., 2013) and ionic transition metal complexes (such as Ru, Ir, and Cd derivatives Costa et al., 2012) typically used for LECs and optoelectronics. Using 100 mL volume autoclaves allows gram scale syntheses of CQDs instead of smaller laboratory amounts.

### Characterization

#### HRTEM of CQDs

**Figure 1:**
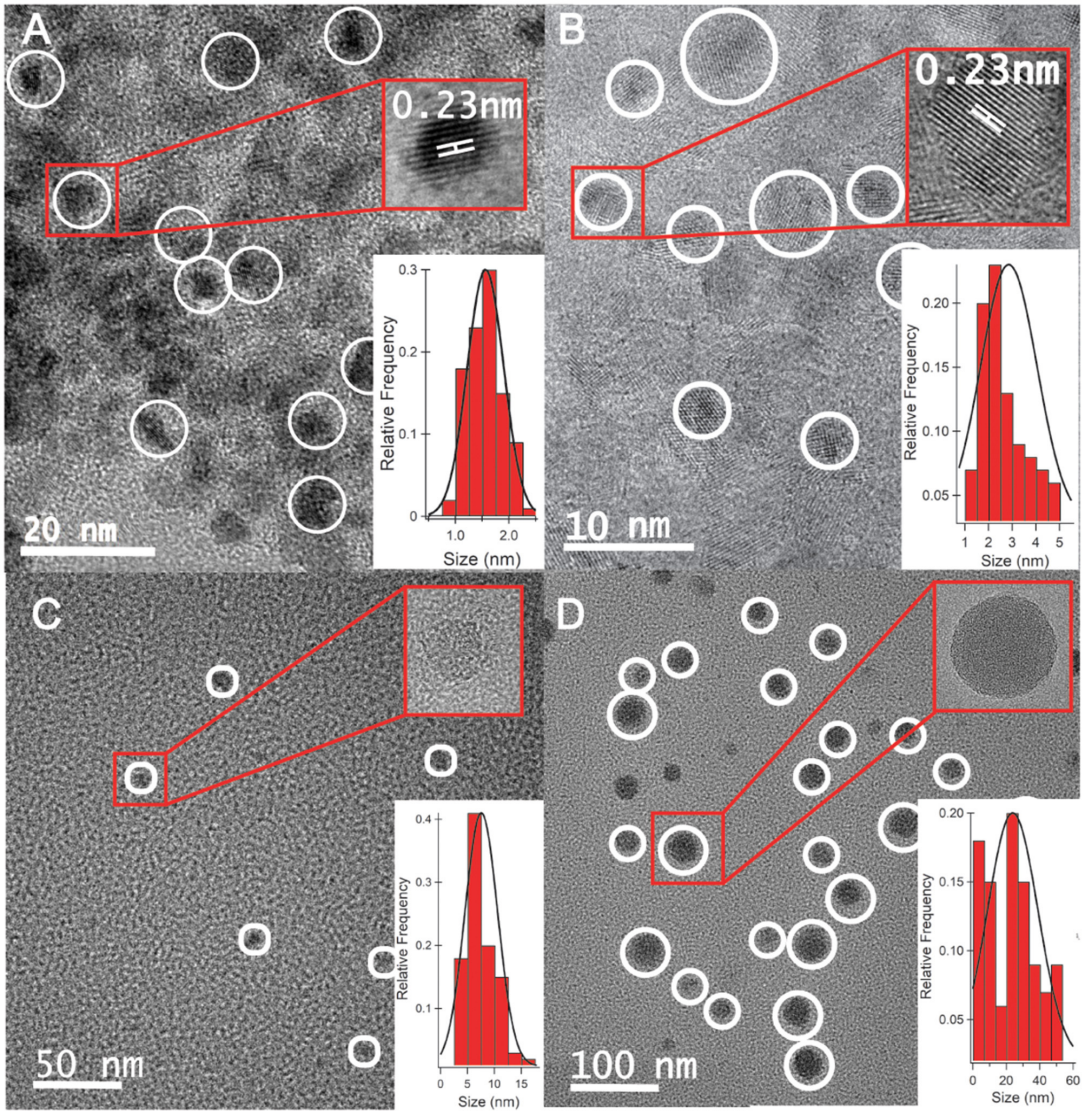
HRTEM analysis of **(A)** CQD10, **(B)** CQD20, **(C)** CQD30, and **(D)** CQD40, each containing a high-resolution image of a single CQD and a histogram of particle sizes fitted with a Gaussian distribution.

High-resolution transmission electron microscopy (HRTEM) images of CQD10 (Figure 1A), CQD20 (Figure 1B), CQD30 (Figure 1C), and CQD40 (Figure 1D) were measured (ca. 120 individual CQDs per synthesis method) and statistical distributions were calculated and fitted to the size distributions. These CQDs displayed average sizes of 1.5 ± 0.3, 2.9 ± 1.2, 7.6 ± 3.1, and 23.8 ± 15.2 nm, respectively. The HRTEM results have revealed a gradual increase in the particle size with augmented volume of the starting solution for the hydrothermal synthesis. The increased carbon precursor appears to increase both the size distribution and particle size, Figures 1A–D. Reaction temperature, time, and concentration were all kept constant in this study yielding unique CQDs. The above results revealed a relationship between volume of CQD precursors (i.e., precursor availability) and size of CQDs produced, leading to a simple way to control the size of particles during hydrothermal syntheses. While using different volumes of the same starting solution, higher volumes created larger CQDs. This may be due to increased autoclave pressure increasing energy available during synthesis or availability of reagents available for CQD synthesis. This finding would be significant, because varying CQD sizes has been shown to change physical and electronic properties (Liu et al., 2019; Wang et al., 2019). This control is highly attractive for light emitting materials of future LEC devices.

The HRTEM insets of each panel in Figure 1 are higher-resolution images of individual CQDs. The insets of Figures 1A,B how 0.23 nm graphene lattice spacing corresponding to a (1,120) graphene lattice plane (Cong et al., 2013). We observed this graphitic nature in CQD10 and CQD20, while the larger CQD30 and CQD40 do not. This could be due to increased availability of carbon precursor favoring a disordered carbon sp^3^ structure, or a heterogeneous mixture of different carbon bonding states. The differences in the carbon bonding of the CQDs has been shown to affect the emission of CQDs (Liu et al., 2019; Wang et al., 2019). Increased graphene nature can cause greater electron delocalization and stabilize the CQDs. The differences in core states observed by HRTEM should provide changes in the emissions of these CQDs, allowing for a platform to optimize the CQDs for electrogenerated chemiluminescence (ECL) for use in optoelectronics. Also, for optoelectronic devices, consistency and packing of CQD films are of the upmost importance since film quality and properties often rely on particle sizes and how they interact (Winkler et al., 2006). For this reason, the size and crystallinity of CQDs are paramount and should greatly affect the optoelectronic performance of the films.

#### FTIR of CQDs

**Figure 2:**
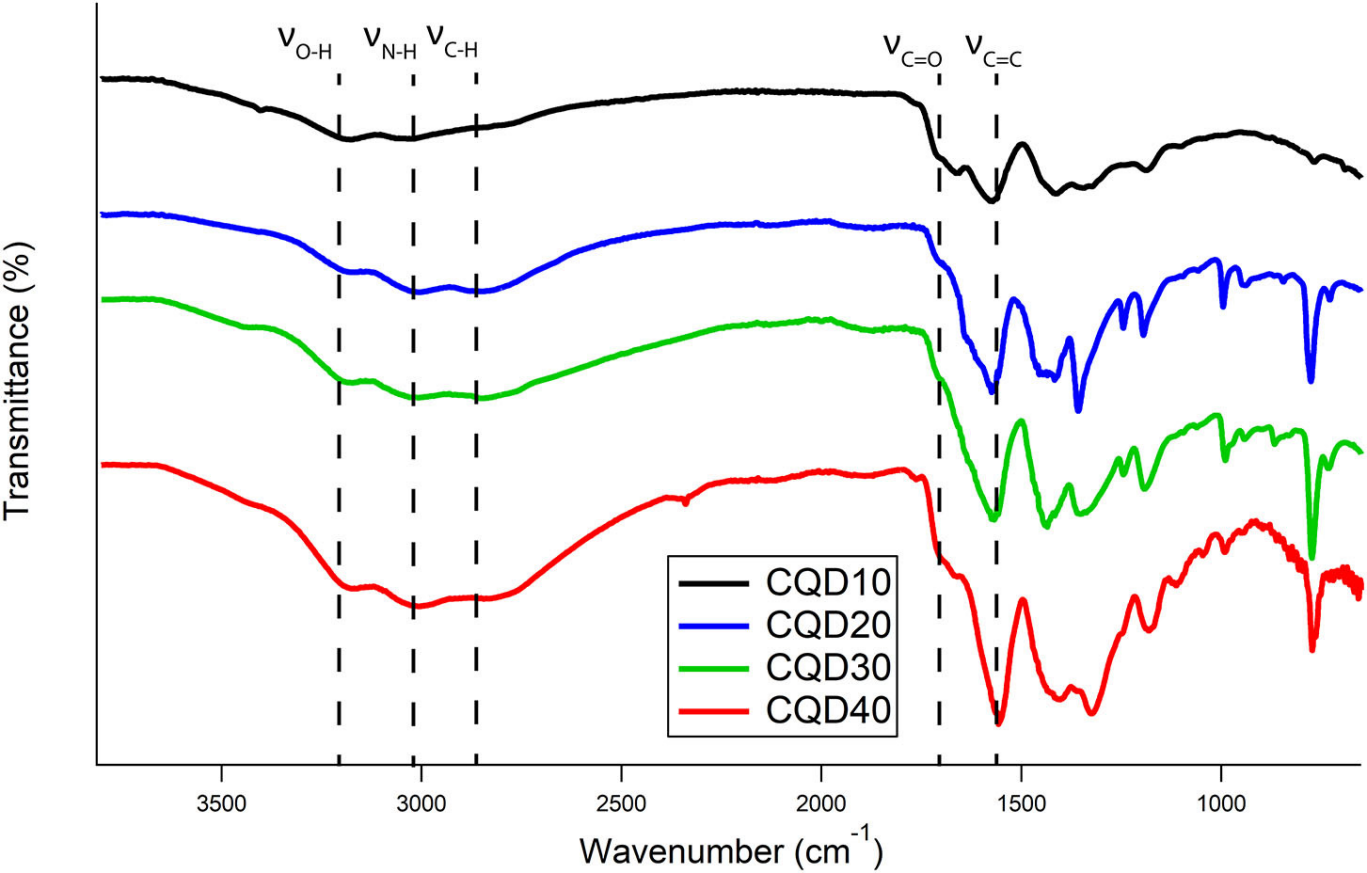
FTIR spectra of CQD10 (black), CQD20 (blue), CQD30 (green), and CQD40 (red).

Absorptions between 3,000 and 3,300 cm^−1^ correspond to O-H and N-H vibrations as demonstrated in Figure 2. The broadness of these peaks indicates that the O-H and N-H are involved in extensive hydrogen bonding between the CQDs. Prolonged freeze-drying at temperatures below 200 K ensure all water was sublimated out from these CQDs, leaving these broad peak identities to be exclusively from hydrogen bonding between CQDs. A broad band at 2,800 cm^−1^ shows C-H stretching characteristic of carbon structures. The bands at 1,700 and 1,600 cm^−1^ were attributed to the vibrational absorption bands of −COOH and C=C, respectively. All FTIR peak assignments agree well with those previously reported studies for CQDs prepared differently (Vinci et al., 2015; Zhang et al., 2017; Dager et al., 2019). No obvious functional group differences between CQDs are demonstrated in Figure 2, which suggests synthesis mechanism is similar, if not the same, between all samples. Although contentious, studies demonstrate evidence for PL and ECL emissions from CQDs originating from surface defects, which are loosely defined as functional groups, oxygen-related disorder-induced localized states and surface defects in carbon structures (Gan et al., 2016; Kroupa et al., 2017). Smaller CQDs have more functional groups per mass compared to larger CQD particles due to the increased surface area per mass. The variation in functional group density might lead to differences in the PL, ECL, and EL emissions of CQD films.

#### Tauc Plot

**Figure 3:**
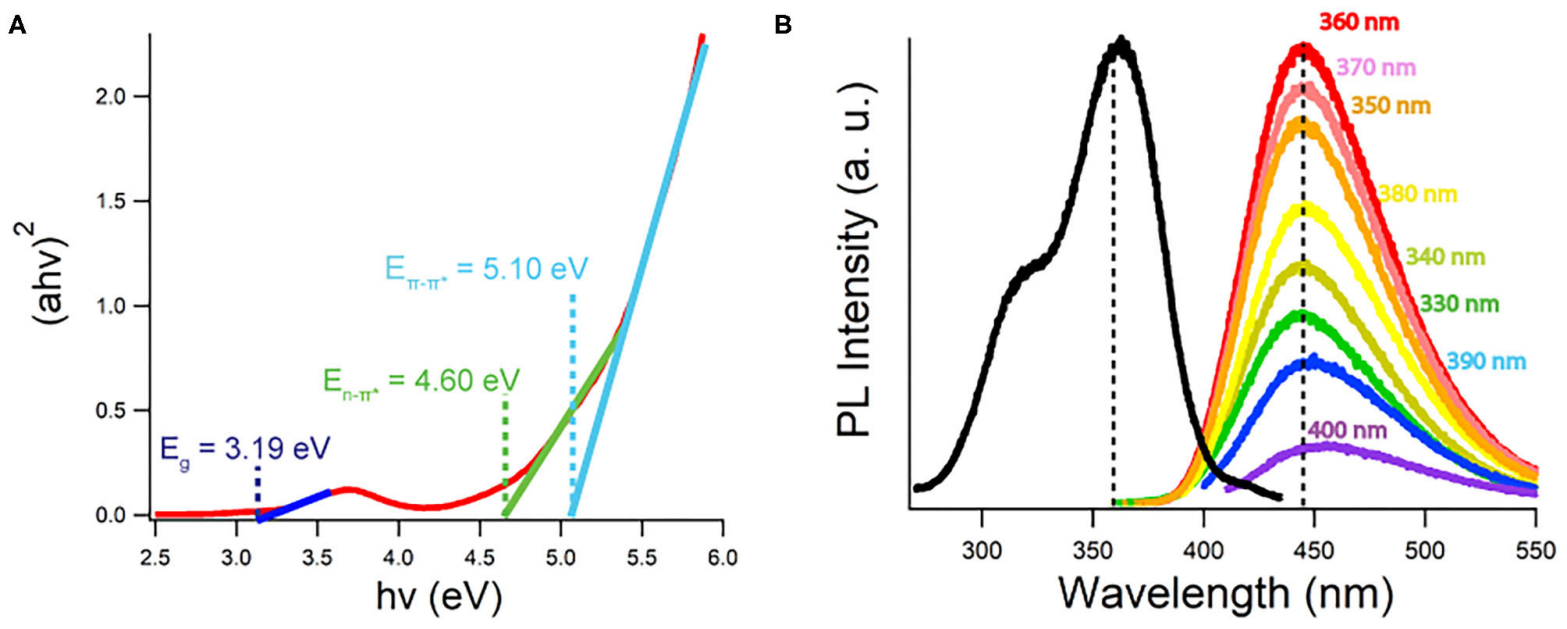
**(A)** Tauc plot generated from 5 g/L CQD10 UV-Vis absorption data created with a direct band gap (*r* = ½). **(B)** Photoluminescence spectra of 5 g/L of CQD10 in water dispersion at excitation wavelengths listed. Black scan measures 440 nm PL intensity vs. excitation wavelengths.

Figure 3A displays a Tauc plot generated from the UV-Vis spectrum of a CQD10 water dispersion at a concentration of 5 g/L. The Tauc plot displays (*ah*ν)^*1/r*^ vs. *hv*, where *a, h*, and ν are absorption coefficients and *r* is the power factor used as a fit for the set of data. The best fit found from Figure 3A is *r* = ½ indicating a direct band gap transformations for the CQD dispersion, aligning well with previously found results on CQDs prepared with hydrothermal methods (Liu et al., 2015; Yang et al., 2015; He et al., 2018). This direct band gap will favor radiative recombination benefitting electrochemiluminescence quantum yield and electroluminescent device efficiencies, as well as achieving strong PL efficiencies. Linear extrapolations to the x-intercept from the low energy side of peaks in the Tauc plots yields specific absorption energies. Tauc plots were constructed for all CQDs, where information gained is summarized in Table 1. The lowest energy absorption in the visible range is due to the band gap of the CQDs. No large deviations from the optical band gap are observed indicating that the state responsible for emission, is shared among all CQDs, despite carbon core state differences. An intrinsic semiconductor E_g_ is illustrated at 3.19 eV corresponding to a blue wavelength absorption. The second peak, common in every Tauc plot generated around 4.60 eV, corresponds to non-bonding electrons in oxygen and nitrogen dopant atoms in the carbon sp^2^ or sp^3^ matrix (He et al., 2018). A significant 0.3 eV shift is noticed comparing the E${}_{\text{n}-\pi^{*}}$ between CQD10 and the other CQDs in this study. CQD20, CQD30 and CQD40's E${}_{\text{n}-\pi^{*}}$ is more pronounced indicating a larger number of dopants in the carbon sp^2^ or sp^3^ matrix (Supplementary Figure 1). As noticed above, the IR spectra showed no significant variations in functional groups between the synthesized CQDs, thus, following this, the difference in absorptions between the CQDs could be from nitrogen and oxygen dopants in the carbon matrix. The small average size of the CQD10 could prevent the development of more complex types of non-bonding electron containing nitrogen moieties (i.e., pyridinic, and pyrrolic moieties). The subtle differences between syntheses parameters may yield more complex nitrogen doping, leading to different absorptions between some CQDs. This absorption difference may be attributed to a higher presence of non-bonding electron moieties found in larger carbon matrices. The third absorption, which was common to all CQDs, was the E${}_{\pi -\pi^{*}}$ transition attributed to the sp^2^ electrons in the carbon matrix (Kroupa et al., 2017).

**Table 1:** Electronic information on all CQDs samples.

**Sample name**	**E_*g*_(eV)**	${\text{E}}_{n-\pi^{*}}$(eV)	${\text{E}}_{{\pi -\pi }^{*}}$(eV)	**Emission λ_PL_ (nm)**	**Φ (% vs. Quinine sulfate)**
CQD10	3.18	4.60	5.10	440	18
CQD20	3.26	4.93	5.60	435	34
CQD30	3.25	4.90	5.63	435	18
CQD40	3.16	4.90	5.60	435	24

#### Excitation Wavelength Dependence of Photoluminescence

By tracking the maximum emission wavelength at 440 nm while varying the excitation wavelength, the black trace in Figure 3B is attained for a 5 g/L CQDs dispersion in Milli-Q water. There appears to be two excitation peaks where the maximum PL emission was achieved by using a 360 nm excitation wavelength. Excitations from 330 to 400 nm and their resulting emissions color-coded are shown in Figure 3B, where excitation wavelengths outside this range were omitted due to negligible light emission. The same emission maximum wavelength was achieved from all excitation wavelengths tested within a reasonable error. This excitation wavelength independence suggests that one emission pathway is responsible for almost all band gap emissions from the CQDs. Despite the size distribution of the CQDs, no large differences were noticed in the PL emission, suggesting a common emission state. Liu et al. has demonstrated that short wavelength emissions originate from core states and long wavelength emissions emit from surface states (Liu et al., 2019). Gan et al. has also summarized recent findings, describing a common hypothesis that CQDs have an emission resulting from 1 to 5 nm sp^2^ carbon centers in all CQD sizes (Gan et al., 2016). Despite large size differences, common emission states existed between all CQDs providing evidence for small sp^2^ light emitting sites dominating PL emissions in these CQDs. It is plausible that the emissions seen in Figure 3B do not resemble surface state emissions or are dominated solely by one surface state emission due to their excitation wavelength independent emissions.

The peak emission wavelength is seen at 440 nm for CQD10 in Figure 3B. Other maximum wavelengths obtained from PL are summarized in Table 1 for all CQDs in this study. The maximum emission wavelengths from all CQDs are similar with small variation centered around 440 nm. Using the International Commission on Illumination (CIE) standards for relating color to light through red, green and blue (RGB) contributions, the PL emission from all CQDs corresponds to a deep blue emission with CIE coordinates of (0.15, 0.09). This shows that large size differences of the CQDs has little effect on the PL wavelength emitted.

PL quantum yields (Φ_PL_) of all CQDs were determined relative to a quinine sulfate standard (C_40_H_50_N_4_O_8_S), chosen for its similar emission wavelength to CQDs at 450 nm. The most efficient emission is from CQD20 with a relative quantum yield Φ_PL_ of 34 %. There was no obvious trend in the emission efficiency of the four prepared CQDs. This shows that large size differences of the CQDs have little effect on the PL efficiency. The intensity of the emission and direct band gap nature of the CQDs, show favorable properties for efficient emission in optoelectronic devices.

### Electrochemistry and Electrochemiluminescence of Carbon Quantum Dots

#### Electrochemistry of CQD Dispersions

**Figure 4:**
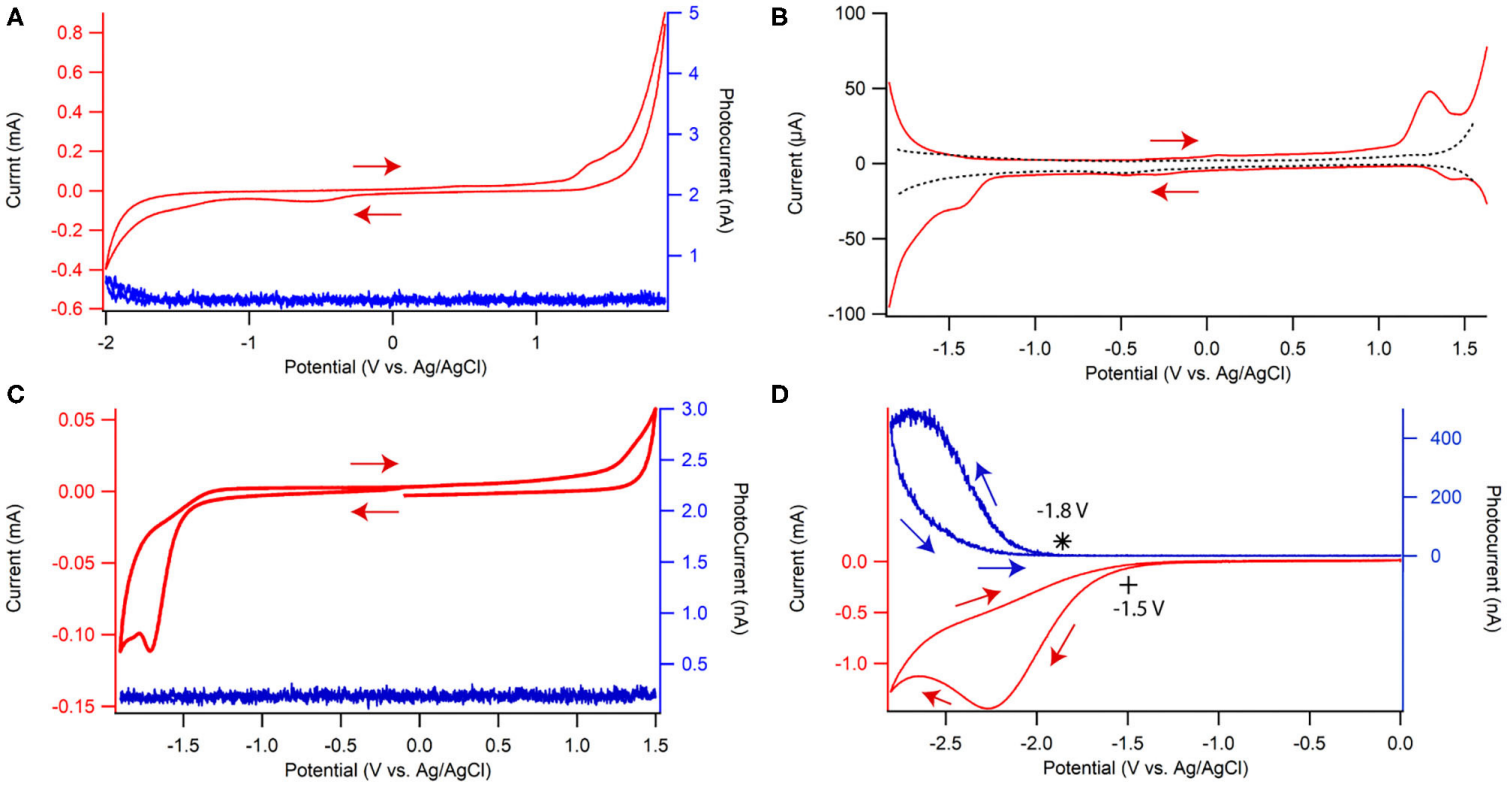
**(A)** Cyclic voltammogram ECL-voltage curve and **(B)** differential pulse voltammograms of 0.1 g/L CQD10 in 0.1 M phosphate buffer solution at pH of 7.5 with 0.1 M KCl as the supporting electrolyte. Cyclic voltammograms (red) and ECL-voltage curves (blue) of a CQD-modified glassy carbon electrode in the same solution without **(C)** and with **(D)** 100 mM K_2_S_2_O_8_, where * denotes the onset of photocurrent during the ECL-Voltage curve. ECL intensity is represented by the photocurrent measured with a photomultiplier tube biased at −750 V.

The electrochemistry of dispersed CQD10 in solution was explored in a supporting electrolyte solution of 0.1 M phosphate buffer solution (PBS) at a pH of 7.5 containing 0.1 M KCl by cyclic voltammetry (CV) and differential pulse voltammetry (DPV) as shown in Figures 4A,B, respectively, with a glassy carbon electrode (GCE) as the working electrode. All potentials are referred to vs. Ag/AgCl. In Figure 4A, a small irreversible peak at −1.7 V and a small slightly more reversible peak can be seen at 1.3 V. To better illustrate these peaks, DPV is shown in Figure 4B where background current is suppressed more than in CV. Comparing the red and black traces, representing a solution with and without dispersed CQD10s, respectively, the redox reaction features of the CQDs are well-displayed in Figure 4B. The cathodic scan shows two irreversible reductions at formal potentials −1.4 V and −1.7 V. All redox reactions, which may be hidden in the charging current seen in the CV in Figure 4A, are very evident in the DPV in Figure 4B. The anodic scan illustrates a slightly more reversible oxidation at a formal potential of 1.3 V. Finding these CQD redox reactions allows for the testing of ECL emission.

The electrochemical gap (EE_g_) between the first reduction peak and the first oxidation peak for CQD10 is found to be 2.65 V. In fact, EE_g_ was determined from converting the peak difference in volts to electron volts (eV) using an elementary charge of 1 for an electron. DPV was performed on all CQDs in this study and the corresponding EE_g_ data is summarized in Table 2. The larger CQDs (CQD30 and CQD40) had larger EE_g_'s likely due to the difference in carbon core. The larger and unordered carbon nature may increase the energy needed for redox reactions in solution due to significant electron transfer barriers between differently ordered states.

**Table 2:** Electrochemical information from differential pulse voltammetry of all CQDs in solution and electrochemiluminescence testing of CQD films in the presence of potassium persulfate.

**Sample name**	**Electrochemical gap (EE_*g*_) (eV)**	**Maximum emission λ_ECL_ (nm)**	**Φ_ECL_ (% vs. ${\text{Ru(bpy)}}_{3}^{2+}$ with 50 mM K_**2**_S_**2**_O_**8**_)**
CQD10	2.65	680	96
CQD20	2.71	670	23
CQD30	3.01	750	2
CQD40	2.90	750	3

To understand the electrochemical behavior of a CQD film, GCEs were modified by casting 10 μg of CQDs, followed by 1 μg of chitosan to prevent CQDs dispersing in electrolyte solution. Chitosan was used to allow the CQD film to interact with solution species, and for its stability in neutral solution pH (pH > 6.5) (Suginta et al., 2013; Wu et al., 2014; Xiong et al., 2014; Yan et al., 2016; Eksin et al., 2017; Gonzalez et al., 2018; Sun et al., 2018; Tashkhourian et al., 2018; Pan et al., 2019; Sisolakova et al., 2019; Yang et al., 2019). CV scans show a strong reduction centered at −1.7 V and a slight oxidation peak at 1.3 V in Figure 4C. The reduction and oxidation peaks match those in the CQD dispersion with larger values probably due to the overall film resistivity increase from chitosan at the solid/electrolyte interface. In polymer films, and more specifically chitosan films, the width of the electrochemical peaks in CVs increases and the current sensitivity toward analytes in the film decreases (Sisolakova et al., 2019). The position of the reduction and oxidation peaks shifts to higher energies relative to analyte in dispersion, owing to the hydrophobic regions of the polymer matrix interacting with the solution and complexing with analytes (Jayaprakash et al., 2017). Despite these matrix effects when using chitosan, analytical responses typically improve for analyte detection and electrochemical stability improves for films, all relative to solution. The above redox peak positions would guide us to perform and understand ECL experiments.

#### ECL in Annihilation Pathway

The light produced during the CV tests involving both the CQDs dispersion and its film are shown by the voltammetric ECL curves in blue in Figures 4A,C. This light typically comes from the relaxation of CQD^*^, which is produced *via* the reaction between CQD^•+^ and CQD^•−^ at the working electrode surface. Negligible ECL as photocurrent was seen in both CQD dispersion (Figure 4A) and film cases (Figure 4C) possibly indicating poor electron transfers between CQD radical cations and anions in the film and solution or the poor stability of one or both these electrogenerated intermediates.

#### Two Half Light-Emitting Electrochemical Cells

To test if the CQD film ECL could be enhanced, coreactants were added. Potassium persulfate (K_2_S_2_O_8_) was added to produce sulfate radical anions that can interact with CQD^•−^ for ECL generation (Figure 4D) while tripropylamine (TPrA) was used to electrogenerate TPrA radicals which can interact with CQD^•+^ for ECL production (Hesari and Ding, 2016). These two coreactants are easily transferred into radicals at close redox potentials to CQDs, producing both highly oxidizing (sulfate radical anion) or reducing (TPrA radical) intermediates in the vicinity of the working electrode for further reactions for light emission. Here, the two coreactants are newly utilized to form two half light-emitting electrochemical cells for testing CQD films as potential lighting layers.

#### ECL of CQD Film/S_2_O${}_{8}^{2-}$ Interface as the First Half Light-Emitting Electrochemical Cell

**Scheme 2:**
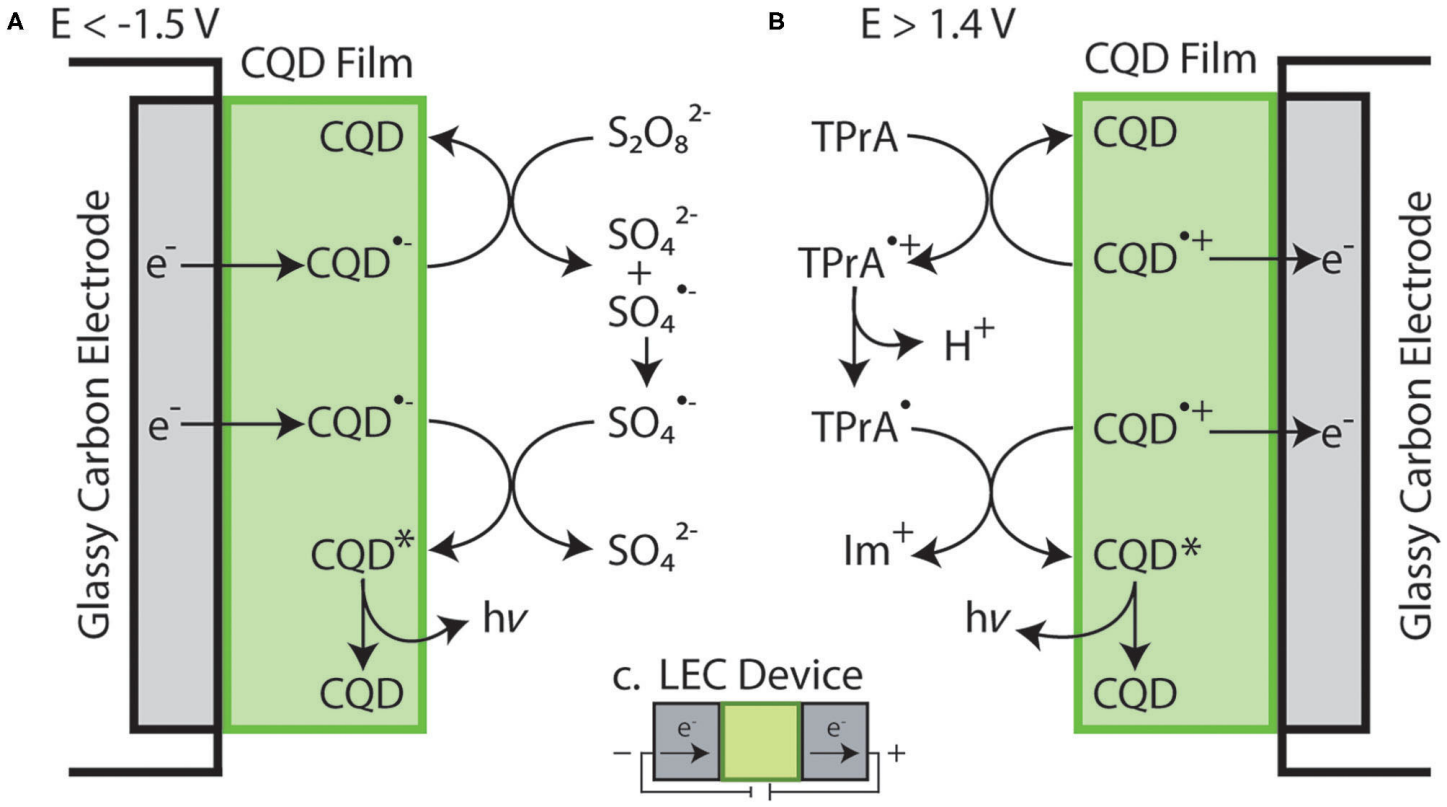
Simplified illustration of reactions occurring at the surface of a CQD film coated on a glassy carbon electrode containing: **(A)** S_2_O${}_{8}^{2-}$ at a potential below −1.5 V vs. Ag/AgCl and **(B)** TPrA at a potential above 1.4 V vs. Ag/AgCl. **(C)** Illustration of the electron flow within a theoretical, fully constructed LEC device.

K_2_S_2_O_8_ is added to solution to test the stability and electron donating nature of CQD^•−^ in film that is formed at −1.70 V, Figure 4D. When biasing potentials more negative than −1.50 V, S_2_O${}_{8}^{2-}$ is electrochemically reduced, then S_2_O${}_{8}^{2-}$ loses SO${}_{4}^{2-}$ to become SO${}_{4}^{•-}$ (Figure 4D). SO${}_{4}^{•-}$ may react with a CQD^•−^ upon generation at −1.70 V to create CQD^*^ that may release energy in the form of light (Scheme 2A). Significant photocurrent as ECL intensity was seen starting at −1.8 V, due to the formation of CQD^*^ as illustrated by the blue trace in Figure 4D. The maximum ECL intensity reaches 530 nA at −2.7 V due to a buildup of both CQD^•−^ and SO${}_{4}^{•-}$ reacting to produce CQD^*^. When the potential was scanned in the reverse direction, the ECL intensity continues to decrease due to the depletion of SO${}_{4}^{•-}$ at the CQD film/solution interface. A blank sample containing only the coreactant and solvent showed negligible amounts of light at the same potentials (Supplementary Figure 2) allowing the light emission to be attributed to the CQD^•−^ reaction with SO${}_{4}^{•-}$ to produce CQD^*^, then giving off light.

Further, the relative efficiency of the ECL emission from the half-cell was determined by finding the charge input and the ECL output for the specific experimental setup and comparing these values to a common commercial ECL emitter system, Ru(bpy)${}_{3}^{2+}$/S_2_O${}_{8}^{2-}$. ECL efficiency tests on this half-cell were performed from solutions with varying concentrations of K_2_S_2_O_8_ yielding an optimized ECL efficiency at a concentration of 50 mM. Other three CQDs were also used to make film electrodes as above and their half-cells were tested as displayed in Supplementary Figure 3. ECL efficiencies were calculated for all CQDs at many S_2_O${}_{8}^{2-}$ concentrations but only the optimized 50 mM concentration results were summarized in Table 2. The smallest CQDs (CQD10) showed the best efficiency and the highest maximum ECL emission. The highest efficiency from CQD10 is roughly the same as the commercially available ECL emitter Ru(bpy)${}_{3}^{2+}$ (96%) (Wallace and Bard, 1979). This half light-emitting electrochemical cell confirms the suitability as the cathodic lighting layer.

For each CQD film electrode, the film thickness is consistent, thus in the smaller CQD10 sample more particles exist on the electrode surface and certainly more functional groups that may produce surface state emissions. Further, only the smaller CQDs (CQD10 and CQD20) showed non-negligible ECL emissions (Supplementary Figure 3), providing further evidence for a surface state ECL emission. CQD10 should be considered for optoelectronics based on its high ECL emitting efficiency.

#### ECL of CQD Film/TPrA System as the Second Half Light-Emitting Electrochemical Cell

Supplementary Figure 4 shows the addition of 50 mM TPrA coreactant to a CQD-modified electrode system during a potentiodynamic scan. The onset of oxidation for the TPrA is roughly at 0.7 V, generating TPrA^+^, then TPrA^•^ through deprotonation. This TPrA^•^ can react with the electrogenerated CQD^•+^ created at 1.4 V, to produce an iminium and an excited state CQD^*^, that can further relax to produce light, Scheme 2B. Despite the highly oxidizing nature of TPrA^•^, negligible photocurrent was observed, indicating CQD^•+^ might not stable enough to react in accepting an electron from TPrA^•^. Furthermore, the electrochemical potentials to generate TPrA^•^ and CQD^•+^ are discrete greatly, which is unfavorable for the CQD^*^ generation, probably due to the instability of both radicals. This half-cell suggests that CQD^•+^ stability and reactivity improvement is required for optoelectronic applications.

#### Comparison of Half-LECs to Full LECs

A simplified illustration of the CQD/S_2_O${}_{8}^{2-}$ and the CQD/TPrA systems are included in Schemes 2A,B, respectively. These individual systems can be thought of as LEC half-cells, where the cathode is Scheme 2A and the anode is Scheme 2B. This similarity between the electrochemical setups and a functioning LEC device are seen in Scheme 2C. The setup in Scheme 2 has CQDs in a film in contact with an electrolyte solution and an electrode. LEC devices are similar, where light emitters are dispersed in a film with electrolyte but are between two electrodes instead of in contact with a solution and an electrode. In fact, ECL provides details about the stability of both ions that are electrogenerated, as well as relative light emission efficiencies, by allowing the testing of multiple coreactants with different reductive and oxidative strengths.

#### Spooling ECL Spectroscopy of the Film/Persulfate Half-Cell

**Figure 5:**
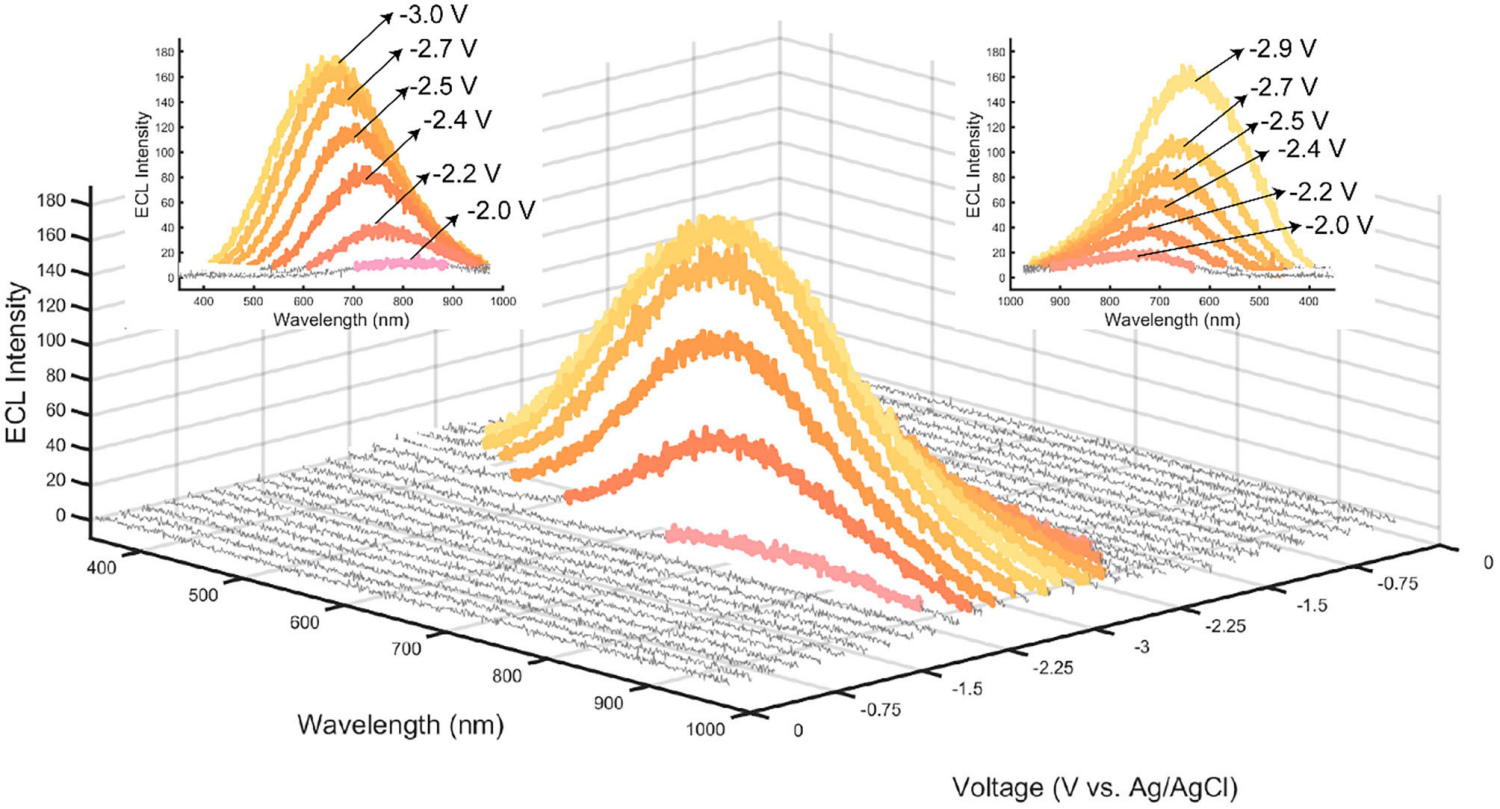
ECL spooling spectra from a film of CQD10 with 50 mM K_2_S_2_O_8_ taken with an exposure time of 2 s, a scan rate of 12.5 mV/s, yielding 120 spectra over a 240 s cyclic voltammogram. Color of individual spectra correspond to the RGB coordinates found by converting spectra using CIE coordinates. Spectra that show negligible light are displayed as gray. Insets show the wavelength of light emitted from a. the forward scanning starting at −2.0 V vs. Ag/AgCl and b. the reverse scan starting at −3.0 V vs. Ag/AgCl.

A potential scanning cycle from 0 to −3.0 V was performed on the CQD film shown in Figure 5, where individual spectra were taken during this potential scan every 2 s (Hesari and Ding, 2016; Shu et al., 2017; Guo et al., 2018). Capturing spectrum during the scan (otherwise known as spooling spectroscopy), enables tracking the evolution/devolution of light emission processes during CV scans. Noticeable light is produced at −2.0 V indicating that at this potential, the electrogenerated SO${}_{4}^{•-}$ and CQD^•−^ react to create CQD^*^, emitting light. The PMT that was used to obtain the ECL-voltage curves is generally more sensitive than a CCD camera and was able to detect light emitted from CQD^*^ at an earlier potential than the CCD camera as illustrated in Figure 4D. Throughout the cathodic potential scan, the wavelength of ECL light slowly blue shifts from a center of 770 nm at −2.0 V, to 650 nm at −3.0 V. This wavelength variation may be due to the coexistence of different oxygen (−OH, −COOH, and −C=O) and nitrogen (−NH_2_, −NRH, and −CONH_2_) functional groups on the CQDs. These functional groups on the surface of the CQDs can contribute to many different surface states, all potentially acting as emissive traps at different potentials, thus changing the wavelength of emission (Bard et al., 2005; Gan et al., 2016; Liu et al., 2019). An increase in the overpotential or driving energy to the CQD films, allows accessing these different energy surface states and may lead to a shorter wavelength or higher energy ECL emission.

The color of each spectrum in Figure 5 illustrates its emission color based on RGB coordinates calculated by a custom MATLAB code produced by our group that uses conventions adopted from the CIE xy chromaticity diagram. By tuning the potential applied to the film, a specific color of light can be achieved between −2.0 and −3.0 V. A wavelength variation is seen during the scan with a slow blue-shift from an emission centered at 790 nm for −2.2 V, to an emission centered at 660 nm for −3.0 V. This wavelength dependency on voltage yields a simple way to achieve multiple colors from one light emitter, providing an attractive emission characteristic for optoelectronic applications.

#### Accumulation ECL Spectra

**Figure 6:**
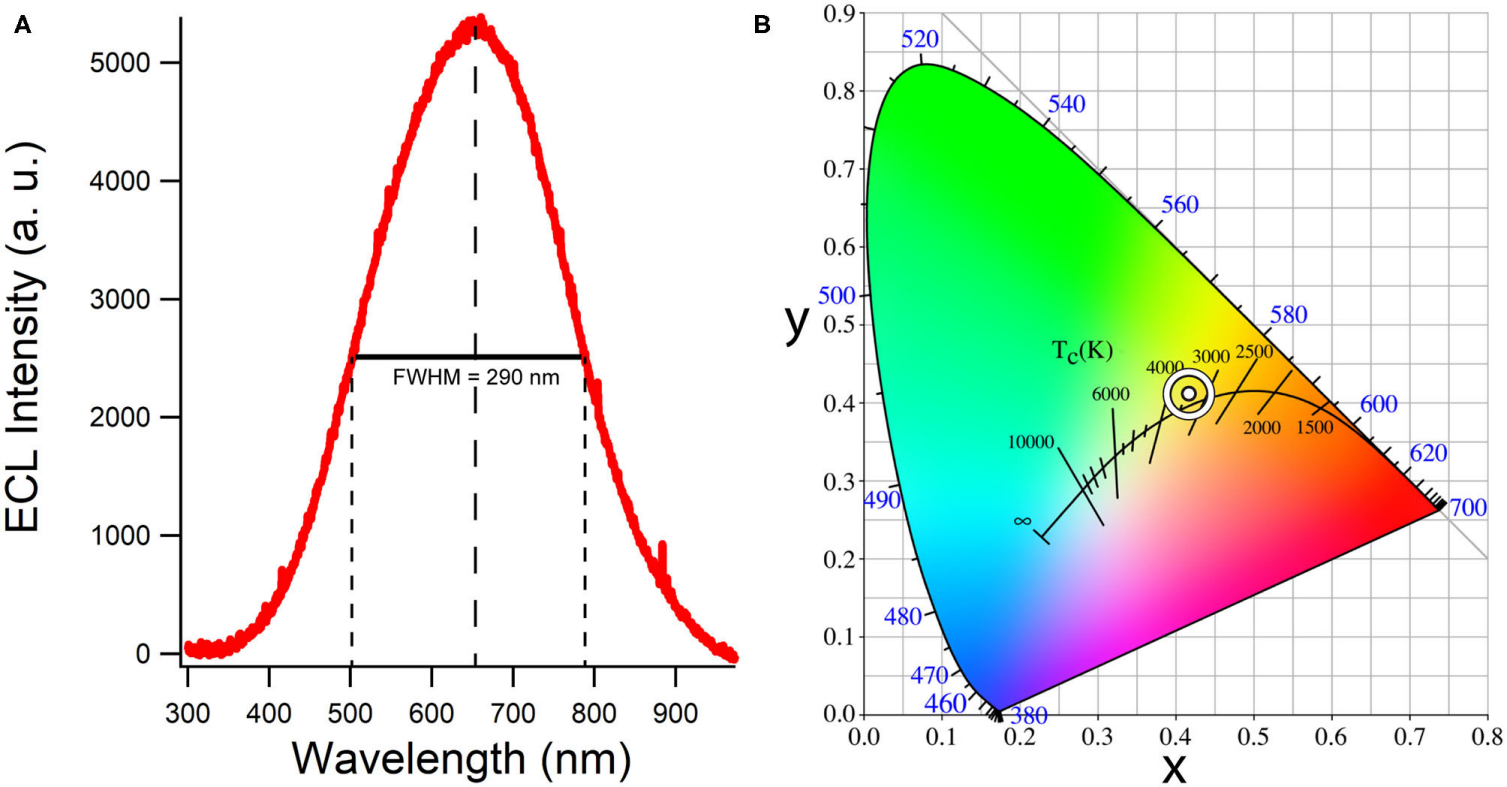
**(A)** A background-subtracted accumulation of all light emitted during the same cyclic voltammogram of the system described in Figure 5. Full-width half maximum is displayed as well as the main emission wavelength. **(B)** The corresponding CIE color space co-ordinates and correlated color temperature.

Figure 6 illustrates an accumulation ECL spectrum during the potential scan shown in Figure 4 over 30 s. The color emitted by the CQD film according to CIE color coordinates is a white light (0.42, 0.41) with a correlated color temperature (CCT) of 3,200 K estimated from the CIE diagram in Figure 6. This CCT value corresponds to a cool, bright and vibrant white and is generally used for most indoor lighting applications. Small CQD films yield relatively strong and efficient white light most likely due to efficient recombination of CQD^*^ generated from CQD^•−^.

## Conclusion

Herein, a protocol to prepare CQDs with controlled size was created from cost effective and readily available precursors. Smaller sized CQDs showed ordered graphitic nature observable in HRTEM but had roughly the same blue PL emission efficiency as the larger CQDs. This shows the PL emissions arising from all CQD particles in this study are similar. As a CQD film, the smallest CQDs had strong white light emission when reacted with 50 mM of the coreactant S_2_O${}_{8}^{2-}$. This emission was as efficient as a typical ECL standard in the same conditions, Ru(bpy)${}_{3}^{2+}$. This ECL emission was centered at 650 nm and gave CIE co-ordinates of (0.42, 0.41) with a CCT of 3,200 K corresponding to a cool bright white light emission. This color of light is ideal for all indoor lighting conditions. The strong efficiency of light conversion, the ease of synthesis and the color temperature of the white light make CQD films a suitable light emitter candidate for LEC applications. Increased surface states per mass of CQDs for small CQDs may capture more holes from SO${}_{4}^{•-}$, allowing a higher ECL efficiency. These ECL tests serve as a simulated LEC half-cells where the stability of each electrogenerated ion can be probed using coreactants and the efficiency of light emission can be simply probed using comparisons to commercial light emitters. Future tests will focus on the implementation of CQDs in LEC devices with improved stability and electron accepting nature of CQD^•+^.

## Data Availability Statement

The raw data supporting the conclusions of this article will be made available by the authors, without undue reservation.

## Author Contributions

JA, RZ, and ZD organized the manuscript. JA and ZD wrote the manuscript. LY, KC, JW, and DL discussed the results. ZD finalized the manuscript. All authors approved this manuscript.

## Conflict of Interest

DL was employed by the company Rosstech Signal Inc.

The remaining authors declare that the research was conducted in the absence of any commercial or financial relationships that could be construed as a potential conflict of interest.
