# Supplementary material for: Efficient White Electrochemiluminescent Emission From Carbon Quantum Dot Films
Source: Front Chem. 2020 Sep 29;8:580022. doi: 10.3389/fchem.2020.580022 (PMC7552666; doi:10.3389/fchem.2020.580022)
Supplement: Supplementary file 1 [file Table_1.docx]

Supplementary Material


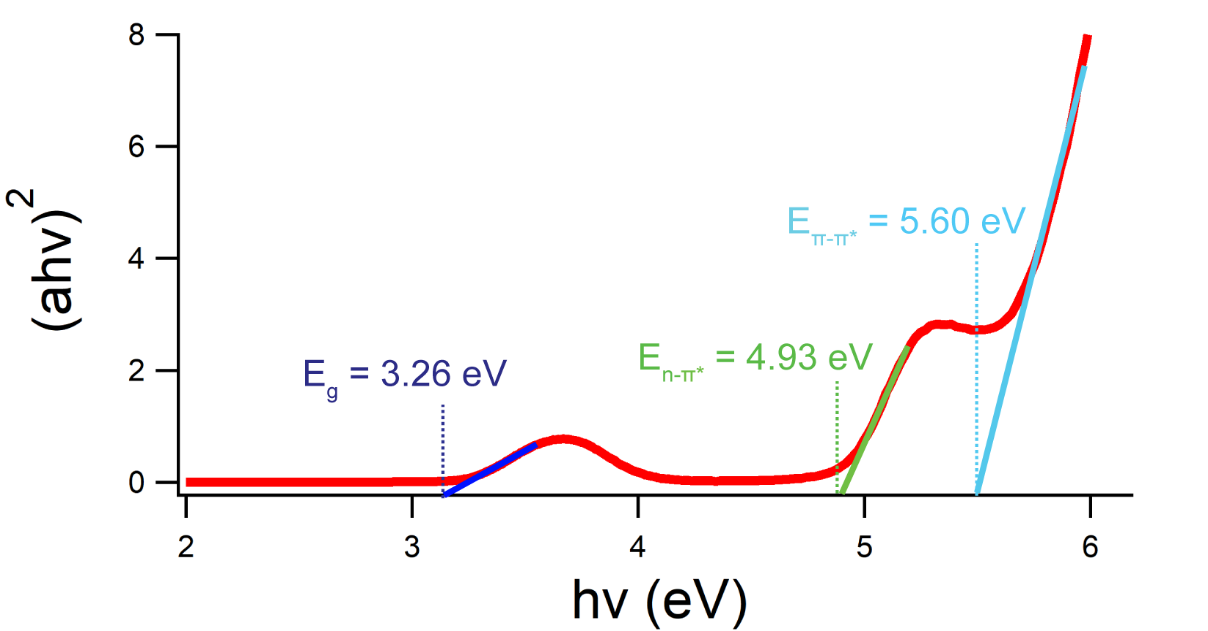


**Supplementary Figure 1**. Tauc plot generated from 0.005 g/L CQD10 UV-Vis absorption data created with a direct band gap (r = ½). All absorptions are labelled and assigned.


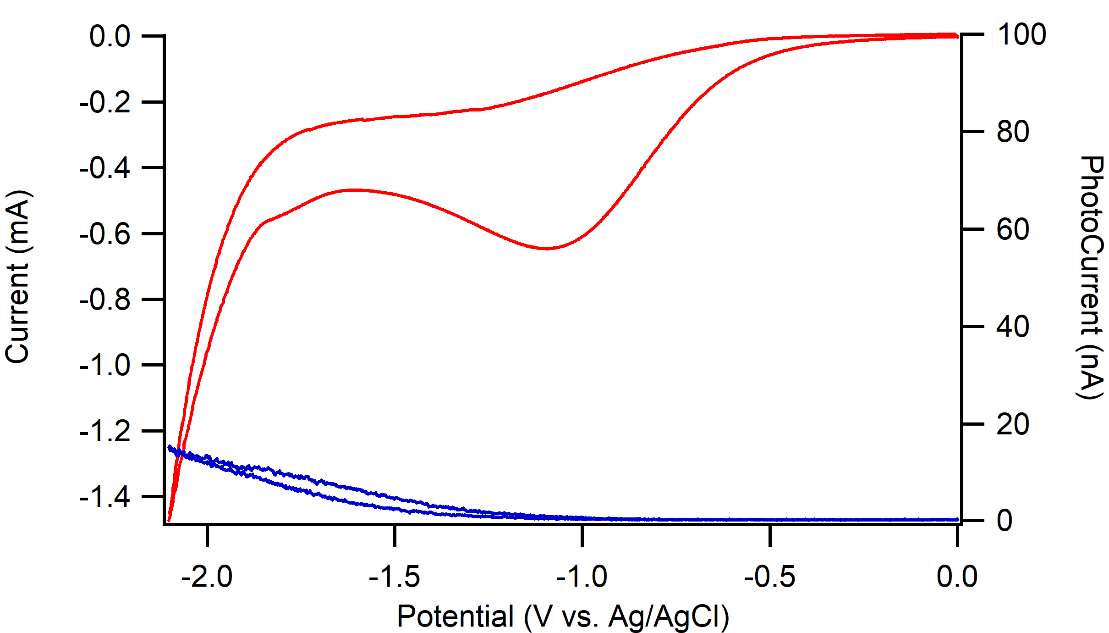


**Supplementary Figure 2**. A bare GCE with 50 mM K_2_S_2_O_8_ in 0.1 M PBS at a pH = 7.5 and 0.1 M KCl.


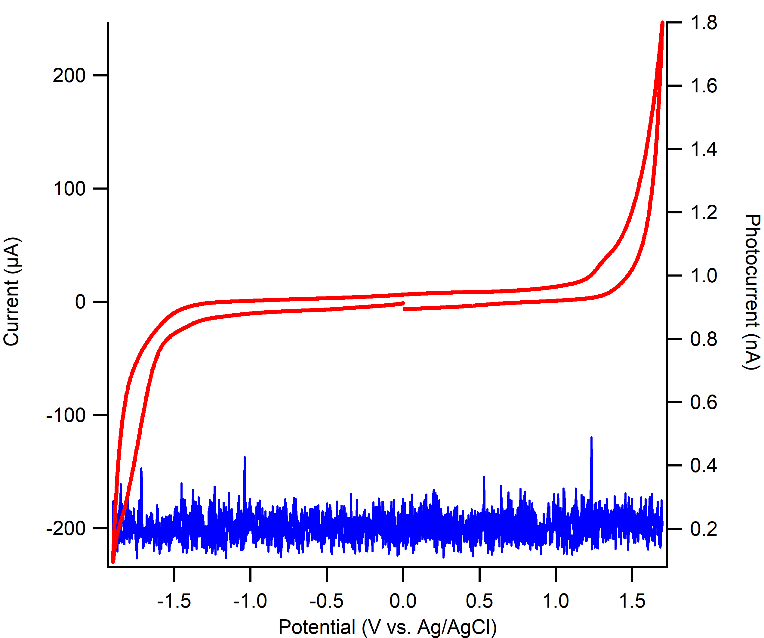


**Supplementary Figure 3**. A cyclic voltammogram of a bare GCE in 0.1 M PBS solution at a pH = 7.5 and 0.1 M KCl.


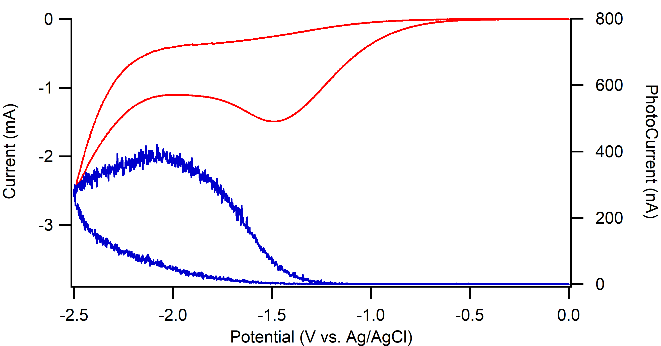

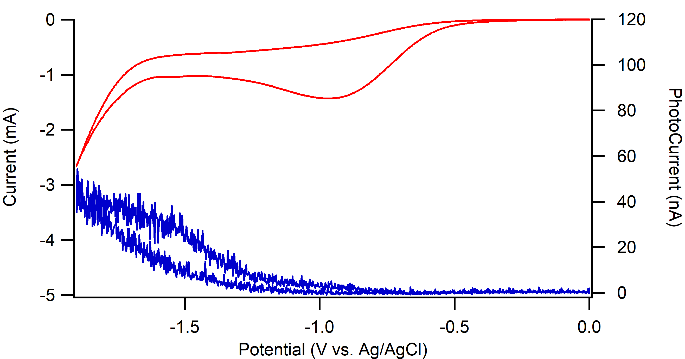


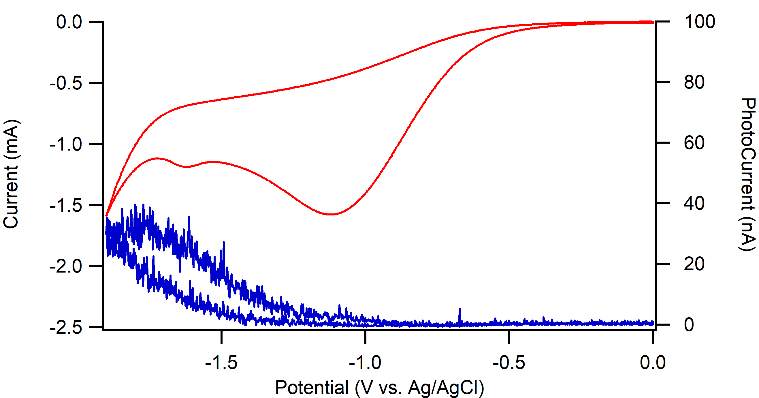


**Supplementary Figure 4**. CQD20 film (top left), CQD30 film (top right), and CQD 40 film (bottom) with 100 mM K_2_S_2_O_8_ in 0.1 M PBS at a pH = 7.5 and 0.1 M KCl.


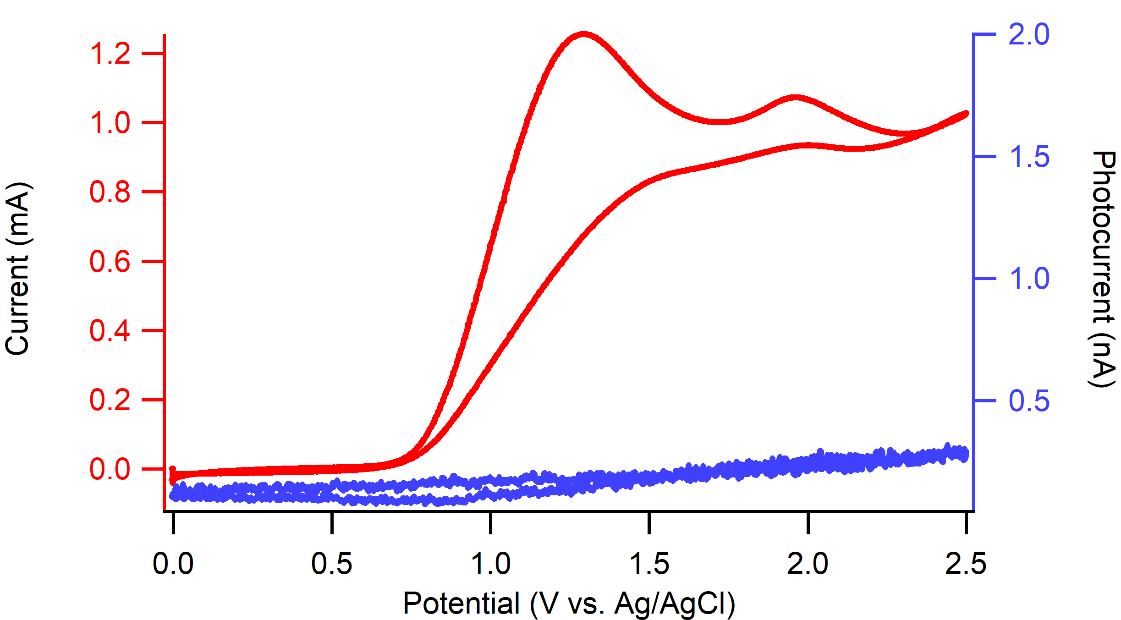


**Supplementary Figure 5**. CQD10 film on a GCE with 0.1 M PBS solution at pH = 7.48 and 0.1 M KCl with 50 mM TPrA coreactant.
